# Examining Age-Adjusted Associations between BMI and Comorbidities in Mongolia: Cross-Sectional Prevalence

**DOI:** 10.3390/healthcare12121222

**Published:** 2024-06-19

**Authors:** Khangai Enkhtugs, Oyuntugs Byambasukh, Damdindorj Boldbaatar, Tumur-Ochir Tsedev-Ochir, Oyunsuren Enebish, Enkhbold Sereejav, Bayarbold Dangaa, Batzorig Bayartsogt, Enkhtur Yadamsuren, Khurelbaatar Nyamdavaa

**Affiliations:** 1 Department of Family Medicine, School of Medicine, Mongolian National University of Medical Sciences, Ulaanbaatar 14210, Mongolia; khangai@mnums.edu.mn; 2 Department of Endocrinology, School of Medicine, Mongolian National University of Medical Sciences, Ulaanbaatar 14210, Mongolia; oyuntugs@mnums.edu.mn; 3 Department of Physiology, School of Bio-Medicine, Mongolian National University of Medical Sciences, Ulaanbaatar 14210, Mongolia; damdindorj@mnums.edu.mn; 4 State Central Third Hospital, Ulaanbaatar 210648, Mongolia; tsch@shastinhospital.mn; 5 Ministry of Health, Ulaanbaatar 14253, Mongolia; oyunsuren@moh.gov.mn (O.E.); enkhbold@moh.gov.mn (E.S.); bayarbold@moh.gov.mn (B.D.); 6 Department of Epidemiology and Biostatistics, School of Public Health, Mongolian National University of Medical Sciences, Ulaanbaatar 14210, Mongolia; batzorig@mnums.edu.mn

**Keywords:** body mass index, obesity, disease risk factors, multimorbidity, health surveillance

## Abstract

(1) Background: This study investigated the association between body mass index (BMI) categories and comorbidities in the context of a developing country, utilizing data from a nationwide health screening in Mongolia. (2) Methods: The study included 181,080 individuals (mean age 47.0 ± 15.3, 42.0% male) from the population-based general health screening. We counted the number of diseases from participants’ medical records based on ICD-10 codes, excluding those categorized under Z00-Z99 and codes indicating acute disorders, as well as individuals classified as underweight. (3) Results: Among study participants, the prevalence of two or more comorbidities was 4.2%. The weight distribution comprised 40.4% normal weight; 37.1% overweight; and 16.9%, 4.4%, and 1.2% in the Class I, II, and III obesity categories, respectively. Comorbidities increased with BMI: normal weight (0.222); overweight (0.255); and Class I (0.290), Class II (0.302), and Class III obesity (0.303), suggesting a dose-dependent likelihood of having multiple diseases. Adjusted linear regression (beta coefficients, 95% CIs) showed increased comorbidity risks in overweight (0.017, 0.013–0.021) and obesity (0.034, 0.030–0.039). Interaction analysis with age revealed a significant effect (*p* < 0.001). While comorbidities tend to increase with higher BMI categories in all age-tertile groups, this association was notably stronger among younger individuals. (4) Conclusions: Obesity is associated with a twofold increase in the prevalence of multiple comorbidities compared to normal weight. Our findings also highlight the critical role of age in the development of multiple diseases, with BMI remaining a significant factor across various age groups, encompassing both younger and older adults.

## 1. Introduction

The global surge in obesity is a pressing public health concern, as evidenced by the World Health Organization (WHO)’s declaration of it as a major epidemic [1]. Over 1.9 billion adults worldwide are overweight, with projections indicating that a majority will be overweight or obese by 2030 [2]. The expanding body of research suggests the broadening understanding of the association between obesity and diverse health conditions. Among the proven links are unequivocal connections to diseases such as type 2 diabetes, cardiovascular diseases, certain cancers, sleep apnea, osteoarthritis, non-alcoholic fatty liver disease, gastrointestinal disorders, depression, and reproductive issues. For instance, a previous review indicates the significant health risks associated with excess body weight, particularly its intricate ties to metabolic diseases [3]. Recent advances in multi-omics studies, as outlined by Zhang et al. [4], reveal the complex mechanisms connecting excess body weight to various comorbidities, highlighting the urgent need for global strategies to address this crisis comprehensively. Tsur and Twig [5] drew attention to the expanding burden of obesity, predicting that one in two US adults will have obesity by 2030, underscoring the need for a holistic understanding of obesity’s impact, considering its intricate association with various comorbidities across the entire clinical spectrum [5]. Recent findings shed light on the global prevalence and time trends of central obesity, providing valuable insights into its multifaceted impact on health [6]. Further, the study by Haththotuwa et al. [7] delves into the worldwide epidemic of obesity, suggesting its far-reaching consequences for health. These collective insights reinforce the urgent need for comprehensive and targeted interventions to mitigate the escalating obesity crisis on a global scale.

Mongolia, as a representative of developing nations, provides a distinctive backdrop for this exploration [8]; the country is facing a burgeoning public health crisis as the prevalence of overweight and obesity escalates [9,10]. Previous studies have delved into this complex issue, shedding light on its multifaceted nature and underscoring the imperative for targeted interventions. Chimeddamba et al. (2016) [11] conducted a previous study on trends in adult overweight and obesity prevalence in Mongolia from 2005 to 2013, revealing a significant upward trajectory based on nationwide non-communicable disease risk prevalence studies. Furthermore, their 2017 investigation [12] into increases in waist circumference independent of weight highlighted a nuanced interplay of factors, with four consecutive Mongolian STEPS surveys indicating a dramatic rise in obesity prevalence from 17.3% to 49.4%. Addressing broader health concerns, Suvd et al. (2002) [13] explored glucose intolerance and associated factors at the national level, emphasizing the significant role of obesity as a major contributor to diabetes. Previous studies in Mongolia have primarily examined the relationship between obesity and individual diseases [13,14,15]. Therefore, it is crucial to understand how obese individuals face increased disease risk, particularly the risk of having multiple disorders.

This study aims to explore the link between body mass index (BMI) categories and multiple comorbidities in a developing country, using data obtained from a nationwide health screening in Mongolia. Through this investigation, we sought to enhance our understanding of the health implications associated with BMI in the context of a developing nation, contributing insights that can inform targeted interventions to address the escalating public health crisis of obesity in such regions.

## 2. Materials and Methods

### 2.1. Study Design and Participants

This study utilized data from a nationwide health screening initiative conducted by the Ministry of Health between 2022 and 2023, covering all regions of Mongolia. Participants were individuals who underwent screening during this one-year period. The screening involved 209,055 adults from all 209 family centers across four rural regions and the capital city. To ensure representativeness, each family center achieved a minimum participation rate of 10%. Exclusion criteria included missing and implausible data, particularly essential variables such as BMI and International Classification of Diseases (ICD) codes (*n* = 184,576). To ensure comprehensive inclusion, each participant needed at least one ICD code, typically Z00.00 (Encounter for general adult medical examination without abnormal findings), even when considered disease-free. After excluding underweight individuals (BMI < 18.5 kg/m^2^) and cases with ICD codes for acute disorders, the dataset comprised 181,080 participants for analysis.

### 2.2. Data Collection

Data extraction was performed from the participants’ health screening records. Variables of interest encompassed demographic information, lifestyle factors, BMI measurements, and diagnostic records.

1. Demographic Information: The collected data encompassed age, gender, education, where education was categorized into two groups: “lower” and “above”. The “lower” category included individuals with no education and those with fewer than 6 years of education. Marital status was divided into “married or cohabitant” and “others”, including individuals who were divorced, widowed, or single. Living area was categorized as individuals residing in either urban areas (city) or rural areas (countryside).
2. Lifestyle Factors: Information on lifestyle choices covered fruit and vegetable consumption, assessed using visual aids. These research materials are familiar to doctors because Mongolia has used the WHO STEPS approach to conduct studies, which have been conducted four times since 2005 [16]. Intake exceeding 5 units was considered to meet the criteria for sufficient consumption [17]. Physical inactivity was defined as not achieving 10,000 steps a day or engaging in no additional sports or leisure activity weekly [18]. Smoking categorization included daily smokers, ex-smokers within the last 6 months, or non-smokers [19]. Alcohol use during health check-ups was evaluated by recording self-reported consumption within the last 30 days [20]. For females, the criterion for presence was defined as having consumed 4 or more drinks on a single occasion in the past 30 days. For males, the criteria included having consumed 5 or more drinks on a single occasion in the past 30 days [20]. The assessment utilized standard pictures of drinks to ensure consistency in understanding and reporting.
3. BMI Measurements: BMI was calculated based on participants’ height and weight, categorizing individuals into normal weight (18.5–24.9 kg/m^2^), overweight (25–29.9 kg/m^2^), and three obesity categories (obese, Class I: 30–34.9 kg/m^2^; obese, Class II: 35–39.9 kg/m^2^; obese, Class III: ≥40 kg/m^2^) [1]. We further categorized normal and overweight individuals as non-obese, while those with a body mass index (BMI) exceeding 30 kg/m^2^ were classified as the obese group.
4. Disease Diagnoses: Each participant was categorized based on ICD codes recorded by healthcare professionals using the ICD-10 classification system. Encounters falling within the Z00–Z99 range were considered indicative of no specific diseases, as these codes denote health examinations and administrative purposes and were not counted as specific diseases. To assess disease burden, participant health screening records underwent a thorough review to tally the total number of diagnosed conditions excluding those with Z codes. Participants were categorized as having multiple comorbidities if they reported three or more diagnosed conditions.

### 2.3. Statistical Analysis

The characteristics of the study population were expressed as means with standard deviations (SDs) and as percentages with numbers according to obesity status. The differences between groups were compared using Student’s *t*-test and Pearson’s chi-square test. BMI categories and disease prevalence rates were calculated.

Age- and gender-adjusted estimation of comorbidities was conducted using general linear model analysis across BMI categories: normal weight, overweight, and obesity classes I, II, and III. Linear and logistic regression analyses assessed the relationship between BMI categories (explanatory variable) and comorbidities (outcome variable). Participants were condensed into three groups (normal weight, overweight, and obesity), merging obesity classes I–III for statistical power. Dummy variables were created, with normal weight as the reference category. Unstandardized beta coefficients with 95% confidence intervals (CIs) and odds ratios with 95% CIs were reported, initially unadjusted, then adjusted for age, gender, socioeconomic factors, and lifestyle factors. Age interaction was examined, leading to the establishment of age tertiles, and separate analyses were conducted for each group. In the regression analysis, the variable representing the total number of diseases was log-transformed to address skewness in the distribution, where a constant value of 1 was added to each observation to avoid issues with zero values, and then the natural logarithm function was applied to the adjusted variable to achieve a more symmetrical distribution.

For all statistical analyses, we used IBM SPSS V.28.0. A statistical significance level was set at *p* < 0.05 for all tests.

## 3. Results

The study comprised a total of 181,080 participants, among whom 42% (76,069) were male. The mean age of the participants was 47.0 ± 15.3 years. In terms of education, 7.8% (14,155) had lower education levels, while a significant 79.0% (143,086) were either married or cohabiting. Approximately 25.3% (45,863) of the participants reported sufficient fruit and vegetable consumption, while a considerable 66.2% (119,889) were physically inactive. Smoking was reported by 19.0% (34,377) of the participants, and alcohol use was observed in 7.6% (13,760) of the population. The mean BMI for the entire population was 26.8 ± 4.7 kg/m^2^.

**Figure 1:**
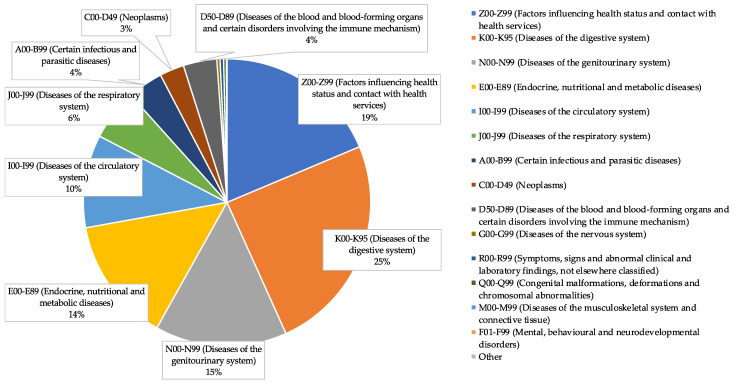
Prevalence of ICD-10 chapters among study participants.

During the screening period, a total of 230,495 ICD codes were registered and classified according to ICD-10 chapters. As shown in Figure 1, the most prevalent comorbidities in our study were diseases of the digestive system (K00–K95), accounting for 34.16% of cases; diseases of the circulatory system (I00–I99), representing 14.52% of cases; and endocrine, nutritional, and metabolic diseases (E00–E89), comprising 19.51% of cases.

Additionally, our analysis revealed that diseases of the circulatory system (I00–I99) commonly co-occurred with endocrine system diseases (E00–E89), highlighting notable patterns of comorbidity within our dataset. For the purpose of the study, we classified individuals based on the presence of multiple comorbidities. Among the total population, 4.2% (7638 individuals) were diagnosed with multiple comorbidities. Statistically significant differences were observed in several characteristics between those with and without multiple comorbidities. Individuals with multiple comorbidities were older (52.5 ± 11.9 years) than those without multiple comorbidities (46.8 ± 15.4 years). A lower percentage of males was found in the group with multiple comorbidities (40.6%) than in the group without (42.1%). A greater proportion of individuals with multiple comorbidities had lower education levels (11.2%) compared to those without multiple comorbidities (7.7%). Moreover, individuals with multiple comorbidities were less likely to report sufficient fruit and vegetable consumption (29.1%) than those without multiple comorbidities (25.2%). They were also more likely to be physically inactive (58.4% vs. 66.6%), to be smokers (19.4% vs. 19.0%), and to have higher alcohol consumption (10.0% vs. 7.5%), all with *p*-values < 0.001 (Table 1).

**Figure 2:**
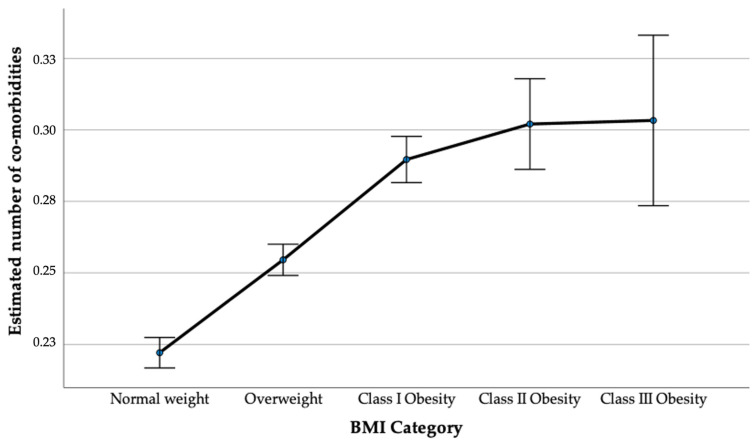
Estimated number of comorbidities across BMI categories.

Among the study participants, 73,220 (40.4%) were classified as normal weight, 67,119 (37.1%) as overweight, 30,607 (16.9%) as Class I obese, 7905 (4.4%) as Class II obese, and 2229 (1.2%) as Class III obese. Age- and gender-adjusted estimated comorbidities were as follows: normal weight had a mean of 0.222 (95% CI: 0.217–0.227), overweight had a mean of 0.255 (95% CI: 0.249–0.260), Class I Obesity had a mean of 0.290 (95% CI: 0.282–0.298), Class II Obesity had a mean of 0.302 (95% CI: 0.286–0.318), and Class III Obesity had a mean of 0.303 (95% CI: 0.273–0.333), indicating a dose-dependent association (Figure 2).

In a linear regression analyses, the dummy variable representing overweight status was associated with an increased risk of comorbidities, with a beta coefficient of 0.029 (95% CI: 0.026–0.033, *p* < 0.001), compared to the reference category of normal weight (Table 2). The dummy variable for obesity demonstrated an even greater association with the presence of diseases, with a beta coefficient of 0.049 (95% CI: 0.045–0.053, *p* < 0.001). Upon adjusting solely for age, the association attenuated but remained statistically significant. However, further adjustments for gender, education, marital status, and lifestyle factors did not substantially alter this association. This suggests that age may indeed play a significant role in the relationship between overweight status and the presence of comorbidities. Further analysis of the logistic regression models indicated that the risk of having multiple diseases increased in the overweight and obesity groups compared to those with normal weight, demonstrating a dose-dependent relationship. This association remained significant and showed minimal change even after additional adjustments (Table 2).

Upon testing the interaction between age and the association of interest, a significant interaction effect was observed (*p* < 0.001). Subsequent analysis involved stratifying the dataset into separate age groups based on tertiles of the dataset’s age distribution. While the regression coefficients remained significant across all age tertiles, they exhibited a decreasing trend from higher to lower age tertiles.

**Figure 3:**
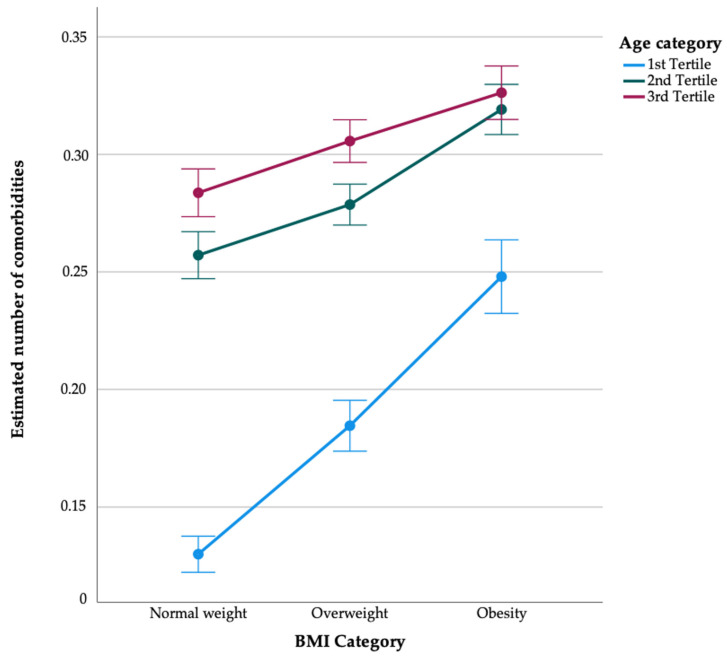
Estimated number of comorbidities across BMI and age categories. Note: Tertiles were defined as follows: Tertile 1 (18–40), Tertile 2 (41–55), Tertile 3 (56–102).

Further examination of comorbidities within each age tertile revealed notable patterns across different BMI categories. In the first age tertile, representing younger individuals, comorbidities increased with higher BMI categories. Specifically, among normal-weight individuals, the mean comorbidities were relatively lower (0.130) than in the overweight (0.185) and obese categories (0.248). This trend suggested a positive association between BMI category and comorbidities, even among younger individuals. This trend was also observed in the second and third age tertiles, although not as linearly as in the younger age tertile, but the dose dependency of BMI category remained evident (Figure 3). These findings suggest the varying impact of BMI categories on comorbidities across different age groups. While comorbidities tend to increase with higher BMI categories, the strength of this association may differ depending on age, with a more pronounced effect observed among younger individuals.

## 4. Discussion

Obesity has emerged as a global health challenge, with its prevalence steadily rising over the past decades [21]. Our study contributes to the growing body of literature by investigating the intricate relationship between obesity and multimorbidity in a representative population in Mongolia. Understanding these associations is crucial for informing targeted interventions and public health strategies tailored to the specific needs of developing countries’ populations grappling with the increasing burden of obesity-related diseases [3,4,5,6,7]. The study included a substantial dataset of 181,080 individuals, providing a robust foundation for examining the impact of varying BMI categories on the prevalence of diseases. The prevalence rates indicated a significant proportion of the population falling within overweight and obese categories. Importantly, the study explored a spectrum of factors including age, gender, education, marital status, lifestyle choices, and other variables, ensuring a nuanced analysis. The findings revealed a dose-dependent relationship between increasing BMI categories and the risk of developing multiple diseases, even after adjusting for confounding variables. Moreover, obesity appears to be linked to a twofold increase in the prevalence of multiple comorbidities compared to individuals with normal weight, while overweight individuals also demonstrate a heightened risk of experiencing such comorbidities. Furthermore, the findings indicate the varying impact of BMI categories on comorbidities across different age groups. While comorbidities tend to increase with higher BMI categories, the strength of this association may differ depending on age, with a more pronounced effect observed among younger individuals.

Comparisons with existing literature underscore the strong association between higher BMI categories and increased risks of various diseases [22,23,24,25,26,27]. Studies by Ward et al. (2019) [22] highlighted elevated chronic disease risks in obesity, while Smith et al. (2020) [23] explored metabolic complexities, and Dorner et al. (2012) [24] examined cardiovascular implications. Additionally, research by Johnson et al. (2018) [25], Garcia-Poma et al. (2020) [26], and Patel et al. (2022) [27] expanded the understanding of obesity’s broader health impacts. Our study, alongside others using hospital-based data, reaffirms the significant link between BMI and health outcomes, particularly in developing-country contexts, contributing valuable insights [28,29,30]. Studies indicate that obese individuals often present with combinations of comorbid conditions such as diabetes, hypertension, and cardiovascular diseases, significantly increasing their health risks. Ward et al. [22] and Liu et al. [29] reported on the frequency and clustering of these comorbidities, while Twig et al. [28] emphasized the elevated risk associated with severe obesity. Additionally, Mutsert et al. [23] and Wang et al. [24] provided insights into the intricate relationships and combined effects of metabolic and cardiovascular disorders in obese individuals. The clustering of metabolic disorders and the elevated risk associated with these comorbidities highlight the critical need for comprehensive management strategies for obesity.

Several studies have highlighted the age-adjusted association between obesity and comorbidities. For instance, Gómez-Acebo et al. [31] found that, after adjusting for age, obesity was more strongly associated with higher comorbidity counts in younger adults than in older ones. Similarly, *Frontiers in Medicine* [32] demonstrated that younger individuals with obesity had a significantly higher risk of developing multiple chronic conditions than their older counterparts when age was taken into account. Research by Song et al. [33] focused on the impact of obesity on various health outcomes and showed that the strength of the association between obesity and comorbidities diminished with increasing age. These findings suggest that the detrimental effects of obesity on health are particularly pronounced in younger populations, underscoring the importance of early interventions.

In Mongolia, renowned for its nomadic heritage and expansive landscapes, a growing health challenge is disrupting traditional norms—the escalating rates of obesity among adults. This research unveils a concerning trend, with 58.1% of Mongolians classified as either obese or overweight, surpassing prevalence rates observed in various other Asian countries. For instance, South Korea reported a lower rate of overweight and obesity, at 36.3%, in 2019 [34]. A comprehensive study on risk factors for non-communicable diseases conducted in China, Mongolia’s neighbor, spanning from 2015 to 2019, revealed an increase in obesity to 16.4% and excess body weight to 34.3% [35]. It is essential to contextualize our findings within the regional characteristics specific to our study area, which is a developing country. Comparing our findings with those from studies conducted in developed countries demonstrates that while the prevalence of obesity-related comorbidities is generally higher in developed countries, the relative risks associated with BMI categories can vary due to differences in healthcare access, lifestyle factors, and genetic predispositions [36]. In developing countries, socioeconomic factors, healthcare infrastructure limitations, and cultural influences significantly impact the prevalence and management of obesity-related comorbidities [37]. In Mongolia, health policymakers and professionals recognize obesity as a growing and significant health problem. Previous studies have primarily focused on the relationship between obesity and individual diseases. Our study, however, specifically examines the risk of having multiple disorders associated with obesity, rather than identifying which specific diseases are linked to obesity. Therefore, future studies should explore which disorders co-occur in the same individuals and are associated with obesity.

The study possesses notable strengths, including the acquisition of representative data from a developing country. As indicated in Table 1, our findings closely replicate those of previous studies, highlighting substantial generalizability, particularly in revealing higher educational attainment among women. However, the cross-sectional design presents a limitation, as it impedes the establishment of causal relationships. Additionally, the preliminary diagnoses from general health screenings have not been validated through confirmation from second-level hospitals, potentially introducing biases into the findings. Moving forward, future research endeavors should aim to explore longitudinal patterns and conduct more in-depth investigations into the intricate interactions associated with specific diseases.

## 5. Conclusions

In conclusion, our study in Mongolia underscores the significant impact of obesity on health, extending beyond geographical boundaries and echoing global research findings. The strong associations observed between higher BMI categories and elevated disease risks highlight the urgent need for targeted interventions and comprehensive public health strategies. Moreover, our findings suggest that even overweight individuals face increased risks of multiple comorbidities, while obesity is associated with a twofold rise in the prevalence of multiple comorbidities. Furthermore, our findings demonstrate that age plays a crucial role in the development of multiple diseases, while BMI remains a significant factor across different age groups, encompassing both younger and older adults. These findings underscore the importance of addressing obesity-related health challenges through effective preventive measures and interventions, especially in developing countries such as Mongolia.

## Figures and Tables

**Table 1:** Characteristics of study population.

Findings	Total	Multiple Comorbidities		*p*-Value
		With	Without	
Total				
Frequency, *n*	181,080	7638	173,442	-
Mean age ± SD, year	47.0 ± 15.3	52.5 ± 11.9	46.8 ± 15.4	<0.001
Gender: male, % (*n*)	42.0 (76,069)	40.6 (3099)	42.1 (72,970)	0.005
Education: lower level, % (*n*)	7.8 (14,155)	11.2 (854)	7.7 (13,301)	<0.001
Married or cohabitant, % (*n*)	79.0 (143,086)	87.2 (6657)	78.7 (136,429)	<0.001
Fruit and vegetables: sufficient, % (*n*)	25.3 (45,863)	29.1 (2222)	25.2 (43,641)	<0.001
Physical inactivity, % (*n*)	66.2 (119,889)	58.4 (4462)	66.6 (115,427)	<0.001
Smoking: smokers, % (*n*)	19.0 (34,377)	19.4 (1480)	19.0 (32,897)	<0.001
Alcohol use, % (*n*)	7.6 (13,760)	10.0 (764)	7.5 (12,996)	<0.001
BMI ± SD, kg/m^2^	26.8 ± 4.7	27.8 ± 4.8	26.7 ± 4.7	<0.001

Data are presented as mean ± SD and percentages (numbers).

**Table 2:** Association between BMI and number of comorbidities.

Analysis and BMI Category	Association of BMI with Multi-Disease Risk							
	Unstandardized Beta Coefficient	95% CI		*p* Value	Odds Ratio	95% CI		*p* Value
		Lower Bound	Upper Bound			Lower Bound	Upper Bound	
Unadjusted								
Normal weight (18.5–24.9)	0 (reference)	-	-	-	1.0 (reference)	-	-	-
Overweight (25–29.9)	0.029	0.026	0.033	<0.001	1.24	1.20	1.27	<0.001
Obese (≥30.0)	0.049	0.045	0.053	<0.001	1.40	1.35	1.44	<0.001
Adjusted for age								
Normal weight (18.5–24.9)	0 (reference)	-	-	-	1.0 (reference)	-	-	-
Overweight (25–29.9)	0.017	0.013	0.021	<0.001	1.15	1.11	1.18	<0.001
Obese (≥30.0)	0.034	0.030	0.039	<0.001	1.27	1.23	1.32	<0.001
Adjusted for age, gender								
Normal weight (18.5–24.9)	0 (reference)	-	-	-	1.0 (reference)	-	-	-
Overweight (25–29.9)	0.017	0.013	0.020	<0.001	1.15	1.11	1.18	<0.001
Obese (≥30.0)	0.036	0.031	0.040	<0.001	1.27	1.23	1.32	<0.001
Adjusted for age, gender, education, marital status, and lifestyle								
Normal weight (18.5–24.9)	0 (reference)	-	-	-	1.0 (reference)	-	-	-
Overweight (25–29.9)	0.016	0.013	0.020	<0.001	1.13	1.10	1.17	<0.001
Obese (≥30.0)	0.035	0.030	0.039	<0.001	1.27	1.23	1.32	<0.001

Regression analysis with dummy exposure variables for disease burden compared to the reference group (normal weight). Data presented as unstandardized beta coefficients with 95% confidence intervals (95% CI). The number of diseases variable was log-transformed.

## Data Availability

The data used to support the findings of this study are available from the corresponding author upon request.
